# Identification of two novel *COL10A1* heterozygous mutations in two Chinese pedigrees with Schmid-type metaphyseal chondrodysplasia

**DOI:** 10.1186/s12881-019-0937-1

**Published:** 2019-12-19

**Authors:** Lingchi Kong, Li Shi, Wenbo Wang, Rongtai Zuo, Mengwei Wang, Qinglin Kang

**Affiliations:** Department of Orthopedic Surgery, Shanghai Jiao Tong University Affiliated Sixth People’s Hospital, 600 Yi-Shan Rd., Shanghai, 200233 People’s Republic of China

**Keywords:** Schmid-type metaphyseal chondrodysplasia (MCDS), *COL10A1*, Type X collagen (α1), Incomplete dominance, C-terminal noncollagenous domain (NC1)

## Abstract

**Background:**

Schmid-type metaphyseal chondrodysplasia (MCDS) is an autosomal dominant disorder caused by *COL10A1* mutations, which is characterized by short stature, waddling gait, coxa vara and bowing of the long bones. However, descriptions of the expressivity of MCDS are rare.

**Methods:**

Two probands and available family members affected with MCDS were subjected to clinical and radiological examination. Genomic DNA of all affected individuals was subjected to whole-exome sequencing, and candidate mutations were verified by Sanger sequencing in all available family members and in 250 healthy donors. A spatial model of the type X collagen (α1) C-terminal noncollagenous (NC1) domain was further constructed.

**Results:**

We found that the phenotype of affected family members exhibited incomplete dominance. Mutation analysis indicated that there were two novel heterozygous missense mutations, [c.1765 T > A (p.Phe589Ile)] and [c.1846A > G (p.Lys616Glu)] in the *COL10A1* gene in family 1 and 2, respectively. The two novel substitution sites were highly conserved and the mutations were predicted to be deleterious by in silico analysis. Furthermore, protein modeling revealed that the two substitutions were located in the NC1 domain of collagen X (α1), which potentially impacted the trimerization of collagen X (α1) and combination with molecules in the pericellular matrix.

**Conclusion:**

Two novel mutations were identified in the present study, which will facilitate diagnosis of MCDS and further expand the spectrum of the *COL10A1* mutations associated with MCDS patients. In addition, our research revealed the phenomenon of incomplete dominance in MCDS.

## Background

Schmid-type metaphyseal chondrodysplasia (MCDS; MIM 156500), the most common type of metaphyseal chondrodysplasia, is an autosomal dominant congenital disorder that is characterized by short stature, waddling gait, coxa vara and bowing of the long bones (primarily involving the femur) [1, 2]. The distinctive clinical features of MCDS also include leg pain, enlarged capital femoral epiphyses or partial metaphyseal abnormalities of the upper limbs [3]. The typical radiographic findings of MCDS are widening and irregularity of the growth plates, especially in the distal femur and proximal tibia [4–6], but only a small percentage of patients have involvement of the upper limbs [7]. In addition, it should be noted that all patients affected with MCDS exhibit normal extraskeletal manifestations [8].

*COL10A1* (MIM 120110), located on chromosome 6q21-q22.3 [9], is closely associated with MCDS when pathogenic variants occur in this gene [10–12]. The product of the *COL10A1* gene is the α1 chain of type X collagen, which composes the type X collagen by forming a homotrimer [5]. Type X collagen is a member of the collagen superfamily of structural macromolecules, which has a unique expression pattern localized to the hypertrophic chondrocytes of growth plate cartilage [13]. The function of type X collagen, as a short-chain minor collagen of cartilage, is to play an important role in fetal chondrogenesis and endochondral ossification [5, 14]. Therefore, specific mutations in *COL10A1* are likely to result in occurrence of MCDS.

To date, over fifty mutations in *COL10A1* have been reported in the Human Gene Mutation Database (http://www.hgmd.cf.ac.uk/ac/) or relevant literature, and 51 variants of those to our knowledge are directly associated with MCDS (Additional file 1) [15, 16]. Based on different mutation types, the mutations in *COL10A1* can be divided into two categories: single amino acid residue lack or substitution resulting from missense mutations or small deletions (Class I) and truncated peptide attributed to nonsense mutations or frameshift mutations (Class II) [17, 18]. Of note, the frequencies of clinical findings and underlying pathogenic mechanisms of the two types are completely different.

The present study describes the phenomenon of incomplete dominance and summarizes several potential genetic patterns in two unrelated Chinese pedigrees with MCDS. Moreover, mutation screening used to support diagnosis was performed in the two unrelated Chinese MCDS families.

## Methods

### Patient families

**Fig. 1:**
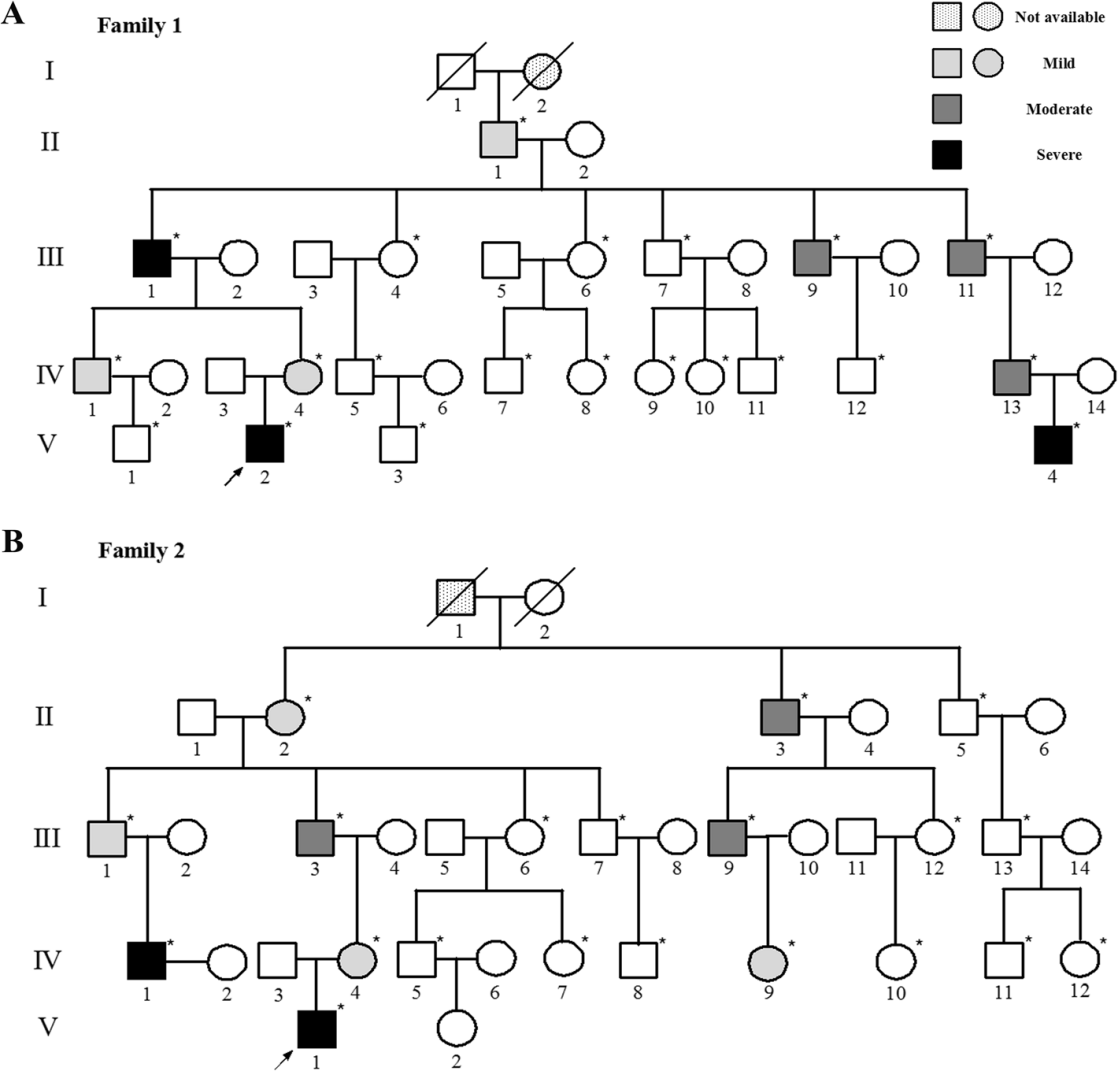
The pedigrees of the MCDS patients. **a** and **b** The grading of disease severity was exhibited using grayscale. Genetic analysis was performed among all patients and available unaffected family members. All individuals who participated in this study were labeled with “*”

Two independent non-consanguineous five-generation families (Fig. 1) containing 19 MCDS patients, identified by two independent orthopedic surgeons, were recruited from the outpatient department of Shanghai Jiao Tong University Affiliated Sixth People’s Hospital (Shanghai, China) when they sought treatment for the probands. All the available patients underwent comprehensive clinical and radiological measurements for diagnosis (all individuals who participated in this study are labeled in Fig. 1). Written informed consent was obtained from all of the participants. The present study was approved by the Ethics Committees of Shanghai Jiao Tong University Affiliated Sixth People’s Hospital.

### Whole-exome sequencing and mutation confirmation

Peripheral blood samples from all of the available family members and 250 unrelated healthy donors aged 18 to 45 years (125 males and 125 females) were collected and stored at − 80 °C. Genomic DNA was extracted from samples using the QuickGene DNA whole blood kit (Kurabo Industries Ltd., Osaka, Japan). A BioAnalyzer 2100 was subsequently employed to assess nucleic acid quantity and quality. We sequenced the whole-exome of all available affected individuals in order to determine the mutated gene resulting in this disorder. The SureSelect Human All Exon 57 Mb Kit (Agilent Technologies, Inc., Santa Clara, CA, USA) and the HiSeq 2000 platform (Illumina, Inc., San Diego, CA, USA) were used to capture the whole-exome sequence following the manufacturer’s protocol. Illumina base-calling software v1.7 was then used to convert the raw image files into 90-base-paired-end reads and all of the variants were validated (Additional file 2).

In the first step, all detected variants were filtered against six single-nucleotide polymorphism databases: the dbSNP144 (hgdownload.cse.ucsc.edu/goldenPath/hg19/database/snp144), the HapMap Project (ftp.ncbi.nlm.nih.gov/hapmap), the 1000 Genomes Project (www.internationalgenome.org), the YanHuang database (yh.genomics.org.cn), the Exome Variant Server (evs.gs.washington.edu/EVS/) and the Exome Aggregation Consortium database (exac.broadinstitute.org), which rejected common single nucleotide polymorphism (SNP) sites (MAF > 1%). In the second step, common single nucleotide variants (SNV) among all family members in each family were of interest but synonymous or intronic variants not located within splice sites were excluded. In the third step, we further checked the conservation of remaining mutations using the UCSC database (https://genome.ucsc.edu/) and the causality of the altered amino acid residues by utilizing Polymorphism Phenotyping version 2 (Polyphen-2; genetics.bwh.harvard.edu/pph2/) and Protein Variation Effect Analyzer (PROVEAN; provean.jcvi.org/index.php/). Finally, the most likely pathogenic candidate mutation was presented in accordance with gene functions and previous studies (Additional file 3).

The identified mutation regions and flanking sequence of the *COL10A1* gene were amplified using a standard polymerase chain reaction protocol to facilitate Sanger sequencing among all available family members and donors. The primers were designed using Primer-3 software (bioinfo.ut.ee/primer.3-0.4.0/). Direct sequencing was performed using the BigDye Terminator Cycle Sequencing Ready Reaction Kit, version 3.1 (Applied Biosystems; Thermo Fisher Scientific, Inc., Waltham, MA, USA), and the sequence was analyzed with an ABI Prism 3130 automated sequencer. Mutation was checked using the Polyphred program (droog.mbt.washington.edu/poly_get.html).

### Protein spatial model construction

The wild-type ribbon structure of the type X collagen (α1) C-terminal noncollagenous (NC1) domain was initially constructed using DeepView and SWISS-MODEL (swissmodel.expasy.org). In addition, to illustrate the positions of the pathogenic mutation and flanking regions, the amino acid residue substitutions were further incorporated into the model.

## Results

### Clinical features

Clinical findings in two MCDS pedigrees are primarily summarized in Table 1. The typical manifestations, including short stature, coxa vara, bowing of the femur and widening and irregularity of the growth plates of the distal femur and proximal tibia observed in X-ray images, were partly found in all patients sooner or later after birth. There were no abnormal perinatal histories or drug abuses in any family members including patients and unaffected individuals. However, the severity of symptoms varied between individuals (Additional file 4). The morbidity rates of males and females in the two families were 52 and 27%, respectively (*P* = 0.19, Fisher’s exact test).

**Table 1:** Clinical and genetic features of affected individuals

Pedigree	Patient	Gender	Age (years old)	Onset age (months old)	Height (SD)	Severity of disease^a^	Pathogenic mutation
Family 1	I:2	Female	Death	12–18	−0.8	Not available	Not available
	II:1	Male	82	Not available	+ 0.4	Mild	c.1765 T > A
	III:1	Male	57	6–8	−2.2	Severe	c.1765 T > A
	III:9	Male	52	~ 12	−1.1	Moderate	c.1765 T > A
	III:11	Male	50	10–12	−1.6	Moderate	c.1765 T > A
	IV:1	Male	33	10–12	−0.4	Mild	c.1765 T > A
	IV:4	Female	28	~ 12	−1.3	Mild	c.1765 T > A
	IV:13	Male	29	6–8	+ 0.1	Moderate	c.1765 T > A
	V:2^#^	Male	2	~ 6	−4.2	Severe	c.1765 T > A
	V:4	Male	4	6–8	−2.5	Severe	c.1765 T > A
Family 2	I:1	Male	Death	Not available	−0.4	Not available	Not available
	II:2	Female	77	~ 12	−0.8	Mild	c.1846A > G
	II:3	Male	72	10–12	−1.5	Moderate	c.1846A > G
	III:1	Male	51	~ 12	−1.1	Mild	c.1846A > G
	III:3	Male	48	10–12	−1.8	Moderate	c.1846A > G
	III:9	Male	49	8–10	−1.9	Moderate	c.1846A > G
	IV:1	Male	24	6–8	−1.5	Severe	c.1846A > G
	IV:4	Female	27	~ 12	−0.7	Mild	c.1846A > G
	IV:9	Female	25	10–12	−1.2	Mild	c.1846A > G
	V:1^#^	Male	1.5	6–8	−4.1	Severe	c.1846A > G

^a^ “Mild” indicates that the patients exhibit i) short stature (mean - 1.5 SD < Height < mean - 0.5 SD) without evident abnormal clinical or radiographic manifestations, or ii) mild genu varum was involved. “Moderate” patients represent that i) mean - 2.5 SD < Height ≤ mean - 1.5 SD, or ii) similar typical radiographic manifestation as illustrated in Additional file 4. “Severe” patients show that i) short stature (Height ≤ mean - 2.5 SD), ii) similar radiographic findings to severe manifestation shown in Additional file 4, or iii) unbearable clinical symptoms, such as arthralgia and restricted motion of the joints. ^#^ Proband of each pedigree

#### Family 1

**Fig. 2:**
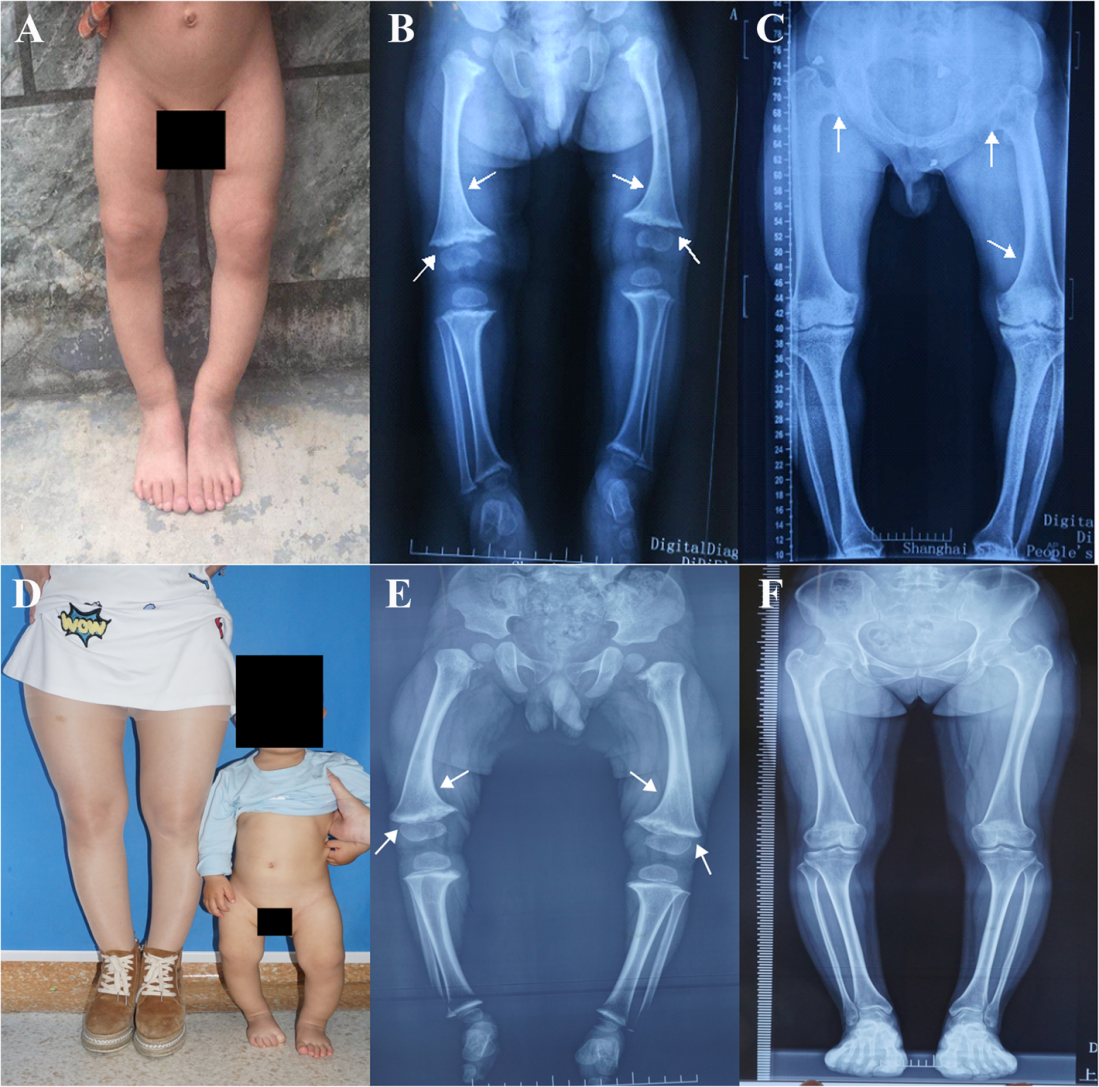
The clinical and radiological features of patients in two affected families. The legs of proband in family 1 exhibited evident “O” shape (**a**) and bowing of the femurs and widening and irregularity of the growth plates of distal femur (white arrows) are shown in X-ray image (**b**). Moreover, the X-ray image of III:1 in family 1 also presented bowing of the femurs, deformity of growth plates and hips (white arrows) (**c**). In family 2, the proband showed deformity of the lower extremities (**d**) and similar radiological presentation (white arrows) (**e**) to proband in family 1. However, the proband’s mother in family 2 exhibited normal appearance in X-ray image (**f**)

Apart from the typical clinical features, proband 1 (V:2), a 2-year-old boy, and his grandfather (III:1) also presented with waddling gait and their affected growth plates appeared “cup”-shaped in X-ray images (Fig. 2a-c). Interestingly, the mother (IV:4) of proband 1 only exhibited relatively short stature without any of the other aforementioned clinical and radiological manifestations. Furthermore, the development and appearance of all other unaffected family members were absolutely normal.

#### Family 2

Proband 2, an 18 month-old boy, had exhibited typical clinical manifestations and slow growth since 6 months after birth (Fig. 2d, e). However, the clinical features of his mother (IV:4) remained mild, similar to IV:4 in family 1 (Fig. 2d, f). Notably, most of the affected members in family 2 were males and male patients were significantly more severely affected than females.

### Genetic analysis

Whole-exome sequencing was followed by systematic screening and bioinformatic analysis. After rejecting common SNP sites (MAF > 1%), SNV were captured. Considering the function of the mutant genes and the results of previous studies, the present study focused on the novel heterozygous missense mutation c.1765 T > A (p.Phe589Ile) (NM_000493.3) in exon 3 of the *COL10A1* gene in family 1 and another novel mutation c.1846A > G (p.Lys616Glu) (NM_000493.3) in the identical region of the *COL10A1* gene in family 2. The two novel mutations were not found in the ExAC and gnomAD databases, so the results further supported the conclusion that the two mutations were novel and rare.

**Fig. 3:**
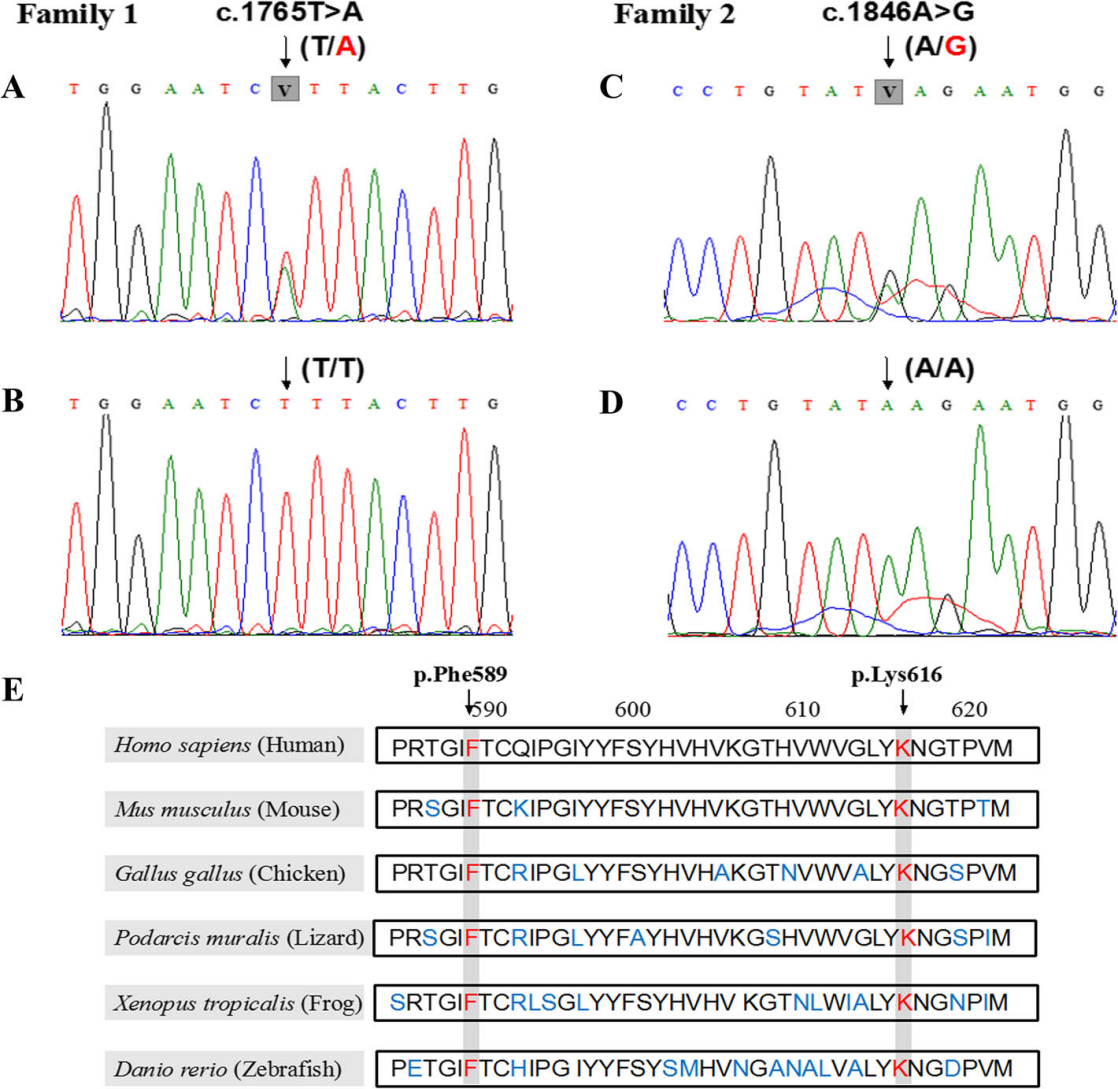
Genetic analysis of patients and unaffected family members. **a** The novel heterozygous mutation site c.1765 T > A (black arrows) of patients in family 1 and **b** corresponding sequence in the other unaffected individuals. **c** The other mutation site c.1846A > G (black arrows) was identified in family 2 patients and **d** wild type site in unaffected family members. **e** Both of the substitution loci p.Phe589 and p.Lys616 were conserved in different species according to the UCSC database

Of note, the changed amino acid residues were highly conserved among several species (Fig. 3e). The Polyphen-2 and PROVEAN scores of the variant c.1765 T > A (p.Phe589Ile) were 1.000 and − 2.69, respectively, which indicated a deleterious function; and the corresponding scores of the other variant c.1846A > G (p.Lys616Glu) were 1.000 and − 1.633.

To confirm the identification of *COL10A1* mutations revealed by whole-exome sequencing, we performed Sanger sequencing of all of the available family members and the 250 healthy donors. In accordance with the results of whole-exome sequencing, the mutations c.1765 T > A (p.Phe589Ile) and c.1846A > G (p.Lys616Glu) were identified in the nine affected individuals in family 1 and in nine affected individuals in family 2, but not in other available unaffected family members or the 250 healthy donors (Fig. 3a-d). Taken together, both of the variants meet the criteria of pathogenic variants according to the American College of Medical Genetics and Genomics Standards and Guidelines (2015).

### Protein structural model

**Fig. 4:**
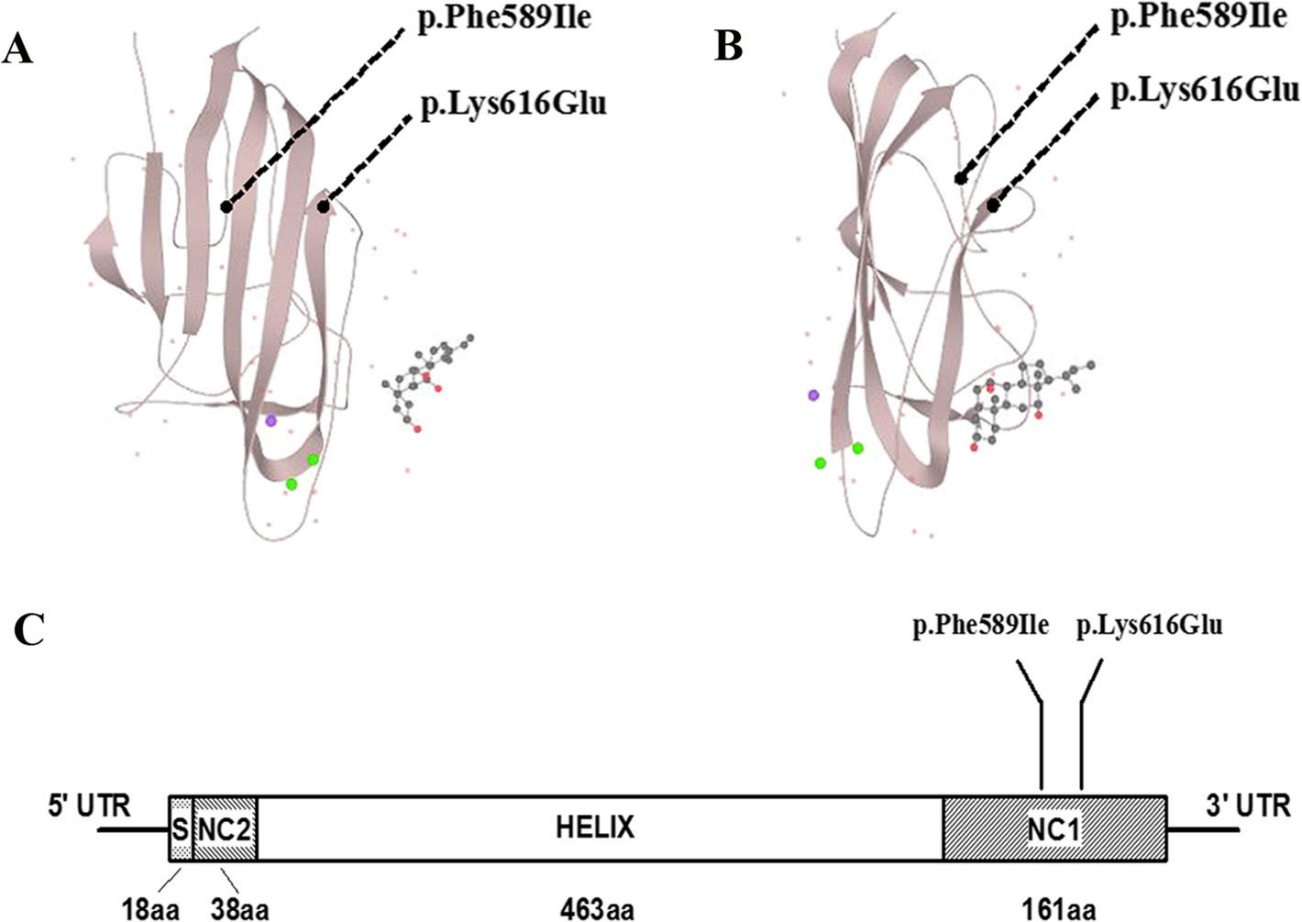
Protein modeling of type X collagen (α1) NC1 domain and stylized structure of collagen X. **a** and **b** As illustrated in the ribbon protein model, both of the novel mutations are located in the NC1 domain of type X collagen (α1). One of the substitutions (p.Phe589Ile) affects a hydrophobic area and the other (p.Lys616Glu) is predicted to affect the surface of the assembled trimer. **c** The stylized structure of type X collagen (α1) is composed of a 18 amino acid signal peptide (S) and a 463 amino acid collagenous domain (HELIX) flanked by a 38-residue NC2 domain and a 161-residue NC1 domain. Furthermore, changes in the present study and most previous variants (Additional file 1) are located in NC1 domain

According to the spatial ribbon structure of the protein, both mutations p.Phe589Ile and p.Lys616Glu were located in the NC1 domain of collagen X (α1) (Fig. 4a, b), where three identical regions interact to form a collagen X homotrimer. Moreover, the replaced wild type residues are conserved among various collagens. It is of note that the two substitutions are located in the hydrophobic area and the surface of the assembled homotrimer, respectively. The mutation p.Phe589Ile weakens the hydrophobicity of the wild type residue, and the other substitution p.Lys616Glu changes the residue site from strongly alkaline to acidic, both of which potentially destroy the interaction between collagen X (α1) peptide chains or between collagen X (α1) peptide chains and other molecules.

## Discussion

There is no doubt that MCDS is an autosomal dominant inherited disorder, resulting from heterozygous mutation of the *COL10A1* gene [4, 10, 17]. In the present study, probands with MCDS and affected family members were identified in two large independent Chinese pedigrees by means of typical clinical findings and genetic analysis. Interestingly, although these individuals suffered from identical disease, there were still differences in the severity of clinical manifestations among all the patients in each family. We attributed the phenomenon of differential performance to incomplete dominance based on the presence of an identical pathogenic mutation in each family, possibly caused by the existence of a modifier gene and environmental differences [19]. Recently, *Forouhan* et al. proposed that ATF6α and ATF6β play important roles in modulating disease severity in MCDS mice by positively or negatively regulating the endoplasmic reticulum stress response [20], which we considered to be the associated mechanism of the incomplete dominance phenomenon. Moreover, carbamazepine, a drug which stimulates intracellular proteolysis and alleviates endoplasmic reticulum stress, effectively reduced the disease severity in the model of MCDS [21]. However, further molecular experiments are needed.

Depending on the differential expressivity of all affected members in two Chinese families (Table 1), we have summarized the following possible rules on the pedigrees affected with MCDS [6, 16]. First, based on the onset age, these patients were characterized by delayed dominance, which only occurred months or even a year after birth, at 6 to 18 months old in this study, rather than at birth. Furthermore, the severity of disease was closely associated with onset age presenting a negative correlation; that is, the earlier the onset age, the more severe the condition. For example, decreased quality of life, including unstable standing or waddling gait was observed in patients whose onset ages were only around 6 months old. Conversely, only short stature without other deformities was exhibited in those whose onset ages were 10 months old or later. In addition, we found that there were possible, but not significant, potential differences in gender susceptibility in MCDS. Moreover, despite the trend that male patients were more severely affected than females, as observed in family 2, we still could not draw a firm conclusion due to the rather small sample size.

The molecular structure of type X collagen is a homotrimer of three X (α1) chains, each consisting of a 463 amino acid Gly-X-Y collagenous domain (COL1) flanked by a 38-residue N-terminal noncollagenous domain (NC2) and a 161-residue C-terminal noncollagenous domain (NC1) (Fig. 4c) [9]. In addition, there is an 18-residue signal peptide ahead of the NC2 domain. To date, a total of 51 mutations of the *COL10A1* gene resulting in MCDS have been reported (Additional file 1). All of the identified mutation sites of *COL10A1* associated with MCDS, including mutations in the present study, are located in the NC1 domain [4, 8, 12, 22–28], except for two missense mutations in the signal peptide and one in the triple helical domain.

As for genotype–phenotype correlations, the varying clinical findings were closely associated with the different molecular mechanisms of pathogenesis between the cases caused by protein-altering variants and those resulting from protein-truncating variants [29]. First, all of the protein-altering variants in *COL10A1* were missense variants, most of which were located in the NC1 domain. The function of the NC1 domain is mainly to assist the folding of the peptide chain so that it can combine to form a homotrimer [5]. Once the NC1 domain becomes impaired, the collagen X (α1) chains are prevented from aggregating and instead form non-functional polymers, which tend to promote harmful accumulation of invalid products and even initiate the endoplasmic reticulum stress response [5, 30]. Meanwhile, the quantity of correctly-folded collagen X is reduced, and therefore functional haploinsufficiency is the most likely cause of the MCDS [14, 31, 32]. These cases caused by missense variants exhibited relatively late-onset ages (generally after 8 months old) and mild or moderate manifestations [4]. Among these variants, the three variants that are not located in the NC1 domain are associated with much later-onset ages and milder manifestations of MCDS, but most of those located in the NC1 domain lead to the more severe forms [25, 27]. On the other hand, *COL10A1* nonsense mutations in cartilage tissue lead to removal of the mutant mRNA by nonsense-mediated mRNA decay (NMD), which is the pathogenic molecular mechanism of nonsense mutations in MCDS [18, 29]. In addition to the functional haploinsufficiency, more complicated molecular mechanisms, such as dominant negative effects were involved in the pathogenesis [8, 29, 33]. The cases caused by *COL10A1* protein-truncating variants showed earlier onset ages (6 months old or earlier) and more severe clinical or radiographic manifestations, such as restricted motion of the joints, compared with those carrying protein-altering variants [6, 8, 28].

In the present study, two novel variants resulted in moderate but differential clinical features in affected individuals. Overall, the genotype–phenotype correlation of cases in this study was consistent with previous reports [4], but the incomplete dominance of phenotype is the first report in MCDS. In terms of pathogenesis, one of the substitutions (p.Phe589Ile) affects a hydrophobic area and the other (p.Lys616Glu) is predicted to affect the surface of the assembled trimer (Fig. 4a, b). The substitution p.Phe589Ile weakens the hydrophobicity of the wild type residue, which is likely to seriously impact the assembly and stability of the hydrophobic channel and thus hinder collagen X trimerization. The other substitution (p.Lys616Glu) changes the residue site from alkaline to acidic, potentially destroying the combination of trimeric collagen X into supramolecular structures within the cartilage pericellular matrix. Together, these biochemical and pathophysiological processes may explain the underlying mechanisms of MCDS in the two present pedigrees. In future, in vitro or in vivo functional studies will be performed to gather more evidence.

## Conclusion

In summary, we identified two novel *COL10A1* heterozygous missense mutations [c.1765 T > A (p.Phe589Ile)] and [c.1846A > G (p.Lys616Glu)] in two relatively large unrelated Chinese pedigrees with MCDS. The genetic analysis facilitated diagnosis of the disease and further expanded the spectrum of *COL10A1* mutations associated with MCDS patients. In addition, our research revealed the phenomenon of incomplete dominance and summarized several potential genetic patterns in the two Chinese pedigrees with MCDS.

## Supplementary information

**Additional file 1: Table S1.** All mutations of *COL10A1* gene resulting in MCDS.

**Additional file 2.** Whole-exome sequencing quality control statistics.

**Additional file 3: Figure S1.** Workflow of bioinformatics and variant filtration process.

**Additional file 4: Figure S2.** Radiographs of mild, moderate and severe cases.

## Data Availability

The datasets generated and analysed during the current study are available in the Mendeley repository, https://data.mendeley.com/datasets/gt6h3nkkrv/1 (DOI: 10.17632/gt6h3nkkrv.1).

## Abbreviations

COL1: Collagenous domain
*COL10A1*: Type X collagen (α1)
MAF: Minor allele frequencies
MCDS: Schmid-type metaphyseal chondrodysplasia
NC1: C-terminal noncollagenous domain
NC2: N-terminal noncollagenous domain
NMD: Nonsense-mediated mRNA decay
