# Supplementary material for: Identification of two novel COL10A1 heterozygous mutations in two Chinese pedigrees with Schmid-type metaphyseal chondrodysplasia
Source: BMC Med Genet. 2019 Dec 19;20:200. doi: 10.1186/s12881-019-0937-1 (PMC6923838; doi:10.1186/s12881-019-0937-1)
Supplement: Supplementary file 1 — Additional file 1: Table S1. All mutations of COL10A1 gene resulting in MCDS. [file 12881_2019_937_MOESM1_ESM.doc]

**Table S1** All mutations of *COL10A1* gene resulting in MCDS.

| Type of mutation | Protein change | Location of mutation | Reference |
| --- | --- | --- | --- |
| Missense mutation | p.Gly18Arg | Signal peptide | [Ikegawa *et al*. (1997)](http://www.ncbi.nlm.nih.gov/sites/entrez?cmd=Retrieve&db=PubMed&list_uids=9067753&dopt=Abstract) |
| Missense mutation | p.Gly18Glu | Signal peptide | [Ikegawa *et al*. (1997)](http://www.ncbi.nlm.nih.gov/sites/entrez?cmd=Retrieve&db=PubMed&list_uids=9067753&dopt=Abstract) |
| Missense mutation | p.Gly288Arg | Triple Helix | [Park *et al*. (2015)](http://www.ncbi.nlm.nih.gov/sites/entrez?cmd=Retrieve&db=PubMed&list_uids=9067753&dopt=Abstract) |
| Missense mutation | p.Thr555Pro | NC1* | [Hasegawa](https://www.ncbi.nlm.nih.gov/pubmed/?term=Hasegawa K[Author]&cauthor=true&cauthor_uid=25678758) *et al*. (2015) |
| Missense mutation | p.Tyr582Asp | NC1 | Bateman *et al*. (2004) |
| **Missense mutation** | **p.Phe589Ile** | **NC1** | **Present study** |
| Missense mutation | p.Cys591Arg | NC1 | [McIntosh *et al*. (1994)](http://www.ncbi.nlm.nih.gov/sites/entrez?cmd=Retrieve&db=PubMed&list_uids=8004099&dopt=Abstract) |
| Missense mutation | p.Gly595Arg | NC1 | [Matsui *et al*. (2000)](http://www.ncbi.nlm.nih.gov/sites/entrez?cmd=Retrieve&db=PubMed&list_uids=10721676&dopt=Abstract) |
| Missense mutation | p.Gly595Glu | NC1 | [Bonaventure *et al*. (1995)](http://www.ncbi.nlm.nih.gov/sites/entrez?cmd=Retrieve&db=PubMed&list_uids=7607655&dopt=Abstract) |
| Missense mutation | p.Tyr597His | NC1 | [Bonaventure *et al*. (1995)](http://www.ncbi.nlm.nih.gov/sites/entrez?cmd=Retrieve&db=PubMed&list_uids=7607655&dopt=Abstract) |
| Missense mutation | p.Tyr597Cys | NC1 | [Sawai *et al*. (1998)](http://www.ncbi.nlm.nih.gov/sites/entrez?cmd=Retrieve&db=PubMed&list_uids=9852679&dopt=Abstract) |
| Missense mutation | p.Tyr598Asp | NC1 | [Wallis *et al*. (1994)](http://www.ncbi.nlm.nih.gov/sites/entrez?cmd=Retrieve&db=PubMed&list_uids=8304336&dopt=Abstract) |
| Missense mutation | p.Ser600Pro | NC1 | [Wallis *et al*. (1996)](http://www.ncbi.nlm.nih.gov/sites/entrez?cmd=Retrieve&db=PubMed&list_uids=8304336&dopt=Abstract) |
| Nonsense mutation | p.Trp611X | NC1 | Bateman *et al*. (2003) |
| Missense mutation | p.Leu614Pro | NC1 | [Wallis *et al*. (1994)](http://www.ncbi.nlm.nih.gov/sites/entrez?cmd=Retrieve&db=PubMed&list_uids=8304336&dopt=Abstract) |
| Nonsense mutation | p.Tyr615X | NC1 | [Mäkitie *et al*. (2005)](http://www.ncbi.nlm.nih.gov/sites/entrez?cmd=Retrieve&db=PubMed&list_uids=16088909&dopt=Abstract) |
| **Missense mutation** | **p.Lys616Glu** | **NC1** | **Present study** |
| Missense mutation | p.Asn617Lys | NC1 | [Bonaventure *et al*. (1995)](http://www.ncbi.nlm.nih.gov/sites/entrez?cmd=Retrieve&db=PubMed&list_uids=7607655&dopt=Abstract) |
| Missense mutation | p.Gly618Val | NC1 | Chan *et al*. (1995) |
| Nonsense mutation | p.Pro620X | NC1 | [Mäkitie *et al*. (2005)](http://www.ncbi.nlm.nih.gov/sites/entrez?cmd=Retrieve&db=PubMed&list_uids=16088909&dopt=Abstract) |
| Nonsense mutation | p.Tyr628X | NC1 | [McIntosh *et al*. (1995)](http://www.ncbi.nlm.nih.gov/sites/entrez?cmd=Retrieve&db=PubMed&list_uids=7749409&dopt=Abstract) |
| Nonsense mutation | p.Tyr632X | NC1 | [Korkko *et al*. (1998)](http://www.ncbi.nlm.nih.gov/sites/entrez?cmd=Retrieve&db=PubMed&list_uids=9452086&dopt=Abstract) |
| Missense mutation | p.Leu644Arg | NC1 | [Bonaventure *et al*. (1995)](http://www.ncbi.nlm.nih.gov/sites/entrez?cmd=Retrieve&db=PubMed&list_uids=7607655&dopt=Abstract) |
| Missense mutation | p.Asp648Asn | NC1 | [Mäkitie *et al*. (2005)](http://www.ncbi.nlm.nih.gov/sites/entrez?cmd=Retrieve&db=PubMed&list_uids=16088909&dopt=Abstract) |
| Missense mutation | p.Asp648Gly | NC1 | [Bonaventure *et al*. (1995)](http://www.ncbi.nlm.nih.gov/sites/entrez?cmd=Retrieve&db=PubMed&list_uids=7607655&dopt=Abstract) |
| Missense mutation | p.Trp651Arg | NC1 | [Pokharel *et al*. (1995)](http://www.ncbi.nlm.nih.gov/sites/entrez?cmd=Retrieve&db=PubMed&list_uids=8554571&dopt=Abstract) |
| Nonsense mutation | p.Trp651X | NC1 | [Mäkitie *et al*. (2005)](http://www.ncbi.nlm.nih.gov/sites/entrez?cmd=Retrieve&db=PubMed&list_uids=16088909&dopt=Abstract) |
| Nonsense mutation | p.Trp651X | NC1 | [McIntosh *et al*. (1995)](http://www.ncbi.nlm.nih.gov/sites/entrez?cmd=Retrieve&db=PubMed&list_uids=7749409&dopt=Abstract) |
| Missense mutation | p.Gln653Pro | NC1 | Bateman *et al*. (2004) |
| Nonsense mutation | p.Gln653X | NC1 | [Woelfle *et al*. (2011)](http://www.ncbi.nlm.nih.gov/sites/entrez?cmd=Retrieve&db=PubMed&list_uids=21360259&dopt=Abstract) |
| Nonsense mutation | p.Tyr663X | NC1 | [Mäkitie *et al*. (2005)](http://www.ncbi.nlm.nih.gov/sites/entrez?cmd=Retrieve&db=PubMed&list_uids=16088909&dopt=Abstract) |
| Nonsense mutation | p.Ser665X | NC1 | [McIntosh *et al*. (1994)](http://www.ncbi.nlm.nih.gov/sites/entrez?cmd=Retrieve&db=PubMed&list_uids=7749409&dopt=Abstract) |
| Nonsense mutation | p.Tyr667X | NC1 | [Mäkitie *et al*. (2005)](http://www.ncbi.nlm.nih.gov/sites/entrez?cmd=Retrieve&db=PubMed&list_uids=16088909&dopt=Abstract) |
| Missense mutation | p.Ser671Pro | NC1 | [Stratakis *et al*. (1996)](http://www.ncbi.nlm.nih.gov/sites/entrez?cmd=Retrieve&db=PubMed&list_uids=8986632&dopt=Abstract) |
| Missense mutation | P.Val677Glu | NC1 | [Park *et al*. (2015)](http://www.ncbi.nlm.nih.gov/sites/entrez?cmd=Retrieve&db=PubMed&list_uids=9067753&dopt=Abstract) |
| Frameshift mutation | p.Phe571fsX605 | NC1 | [Ikegawa *et al*. (1997)](http://www.ncbi.nlm.nih.gov/sites/entrez?cmd=Retrieve&db=PubMed&list_uids=9067753&dopt=Abstract) |
| Frameshift mutation | p.Thr590fsX604 | NC1 | [Wallis *et al*. (1996)](http://www.ncbi.nlm.nih.gov/sites/entrez?cmd=Retrieve&db=PubMed&list_uids=8304336&dopt=Abstract) |
| Frameshift mutation | p.Val603fsX609 | NC1 | Higuchi *et al*. (2016) |
| Frameshift mutation | p.Tyr615fsX621 | NC1 | [Mäkitie *et al*. (2005)](http://www.ncbi.nlm.nih.gov/sites/entrez?cmd=Retrieve&db=PubMed&list_uids=16088909&dopt=Abstract) |
| Frameshift mutation | p.Thr619fsX672 | NC1 | Warman *et al*. (1993) |
| Frameshift mutation | p.Pro620fsX625 | NC1 | [McIntosh *et al*. (1995)](http://www.ncbi.nlm.nih.gov/sites/entrez?cmd=Retrieve&db=PubMed&list_uids=7749409&dopt=Abstract) |
| Frameshift mutation | p.Pro620fsX621 | NC1 | [McIntosh *et al*. (1994)](http://www.ncbi.nlm.nih.gov/sites/entrez?cmd=Retrieve&db=PubMed&list_uids=7749409&dopt=Abstract) |
| Frameshift mutation | p.Pro620fsX621 | NC1 | [Wallis *et al*. (1996)](http://www.ncbi.nlm.nih.gov/sites/entrez?cmd=Retrieve&db=PubMed&list_uids=8304336&dopt=Abstract) |
| Frameshift mutation | p.Tyr623fsX673 | NC1 | Dharmavaram *et al.* (1994) |
| Frameshift mutation | p.Leu633fsX678 | NC1 | Cammarata-Scalisi *et al.* (2019) |
| Frameshift mutation | p.Gln635fsX645 | NC1 | [Park *et al*. (2015)](http://www.ncbi.nlm.nih.gov/sites/entrez?cmd=Retrieve&db=PubMed&list_uids=9067753&dopt=Abstract) |
| Frameshift mutation | p.Ala636fsX676 | NC1 | [Bonaventure *et al*. (1995)](http://www.ncbi.nlm.nih.gov/sites/entrez?cmd=Retrieve&db=PubMed&list_uids=7607655&dopt=Abstract) |
| Frameshift mutation | p.Thr645fsX647 | NC1 | [Wallis *et al*. (1996)](http://www.ncbi.nlm.nih.gov/sites/entrez?cmd=Retrieve&db=PubMed&list_uids=8304336&dopt=Abstract) |
| Frameshift mutation | p.Ala657fsX667 | NC1 | [Park *et al*. (2015)](http://www.ncbi.nlm.nih.gov/sites/entrez?cmd=Retrieve&db=PubMed&list_uids=9067753&dopt=Abstract) |
| Frameshift mutation | p.Glu666fsX677 | NC1 | Goyal *et al.* (2019) |
| Frameshift mutation | p.His669ThrfsX8 | NC1 | Hu *et al*. (2015) |
| Frameshift mutation | p.Val677fsX686 | NC1 | Zhu *et al*. (2011) |
| Complex rearrangements | p.Gly579fsX611 | NC1 | [Mäkitie *et al*. (2010)](http://www.ncbi.nlm.nih.gov/sites/entrez?cmd=Retrieve&db=PubMed&list_uids=16088909&dopt=Abstract) |

* “NC1” indicates a 161-residue C-terminal noncollagenous domain of α1 (Collagen X) chains.
