# Supplementary material for: Identification of two novel COL10A1 heterozygous mutations in two Chinese pedigrees with Schmid-type metaphyseal chondrodysplasia
Source: BMC Med Genet. 2019 Dec 19;20:200. doi: 10.1186/s12881-019-0937-1 (PMC6923838; doi:10.1186/s12881-019-0937-1)
Supplement: Supplementary file 3 — Additional file 3: Figure S1. Workflow of bioinformatics and variant filtration process. [file 12881_2019_937_MOESM3_ESM.ppt]

## Slide 1
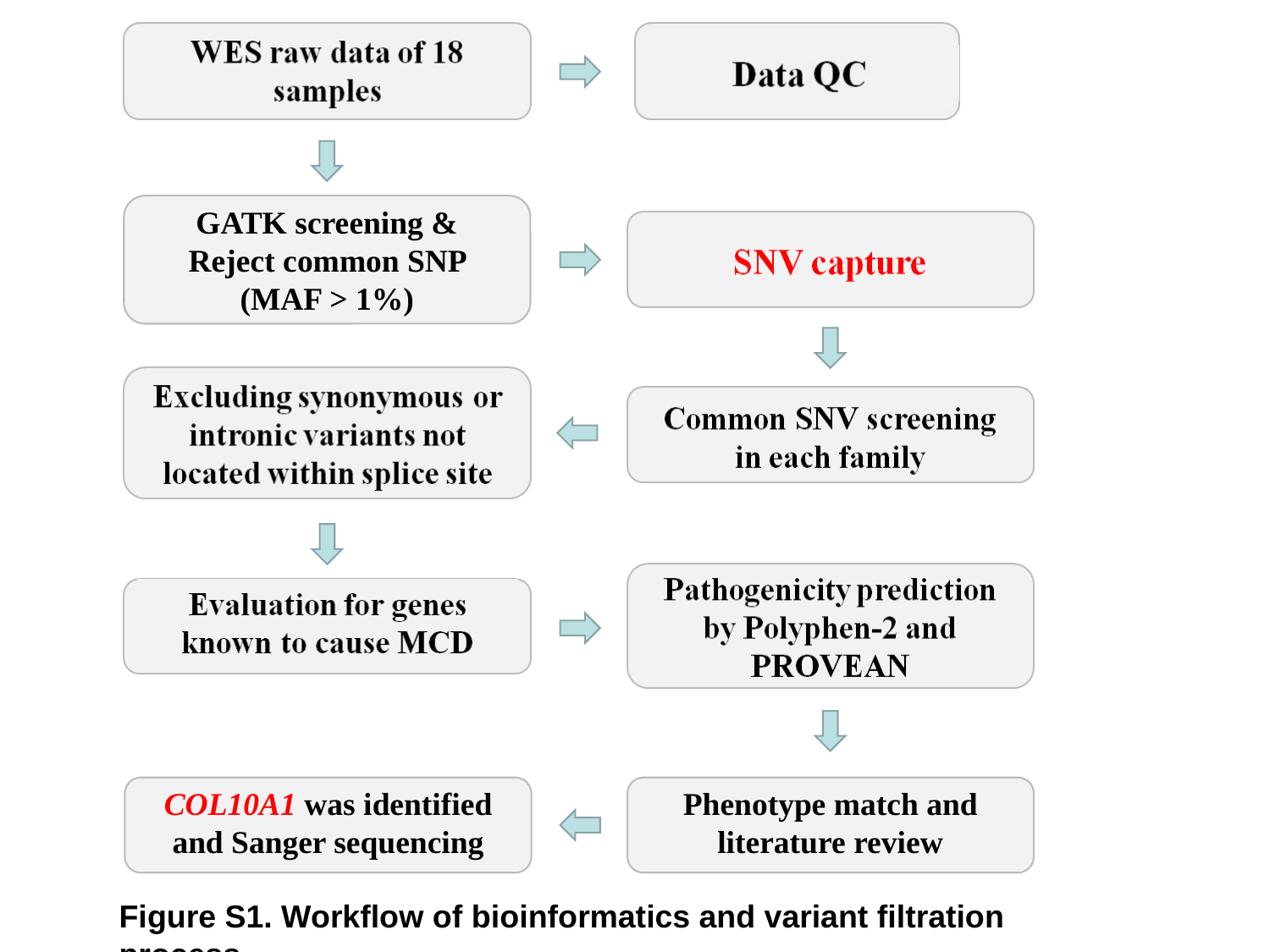

GATK screening &
Reject common SNP (MAF > 1%)
COL10A1 was identified and Sanger sequencing
Phenotype match and literature review
Figure S1. Workflow of bioinformatics and variant filtration process
