# Supplementary material for: Identification of two novel COL10A1 heterozygous mutations in two Chinese pedigrees with Schmid-type metaphyseal chondrodysplasia
Source: BMC Med Genet. 2019 Dec 19;20:200. doi: 10.1186/s12881-019-0937-1 (PMC6923838; doi:10.1186/s12881-019-0937-1)
Supplement: Supplementary file 4 — Additional file 4: Figure S2. Radiographs of mild, moderate and severe cases. [file 12881_2019_937_MOESM4_ESM.doc]

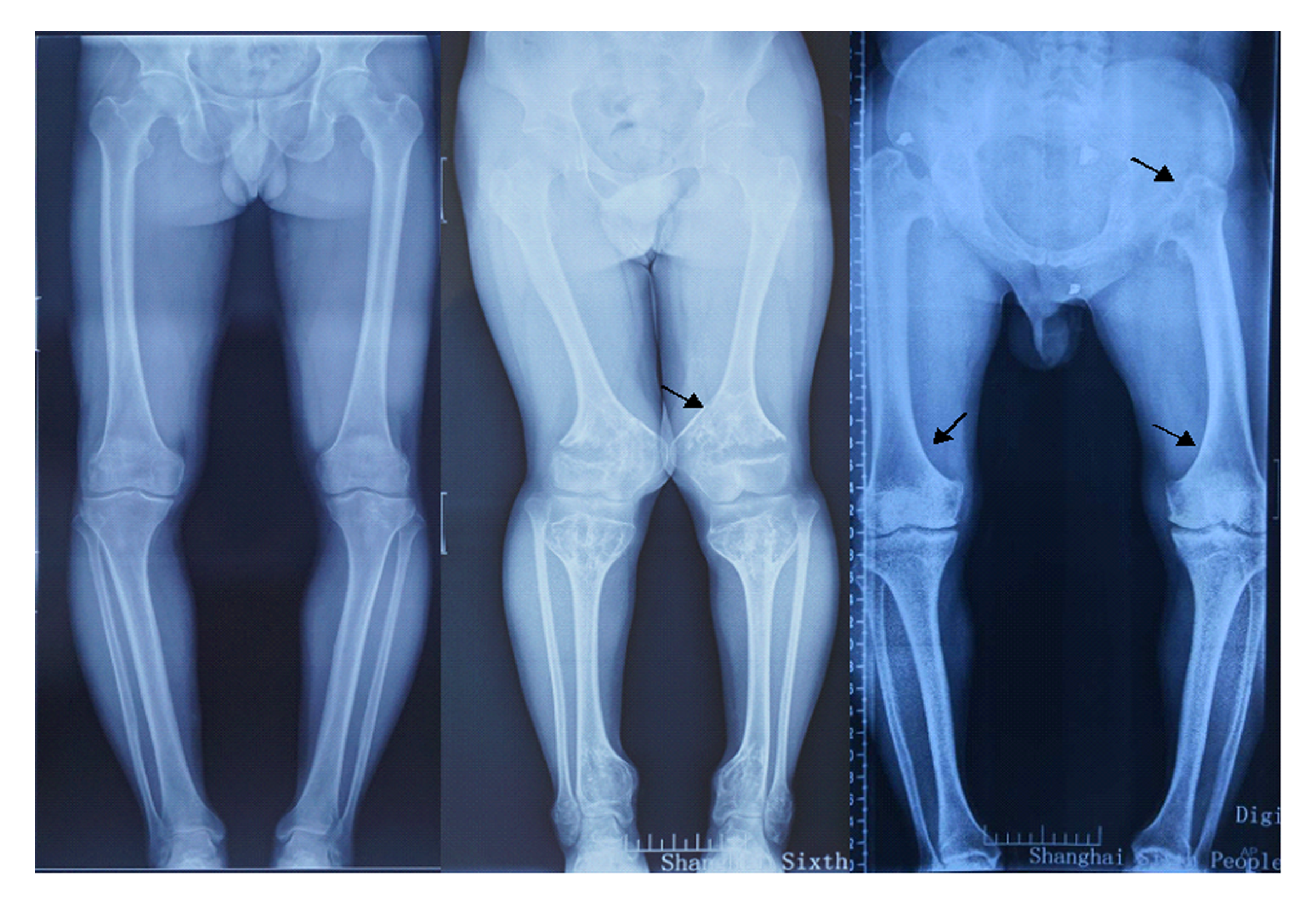


**Figure S2.** Radiographs of mild (left), moderate (middle) and severe (right) cases. The radiographs of mild patients showed no obvious deformity, but mild genu varum may be involved. Enlarged femoral or tibial growth plates and coxa vara were observed in moderate cases. In addition to these, severe cases presented more abnormality, such as bowing of the femurs and hip dyskinesia.
